# Effects of different conditioning activities performed after soccer-specific warm-up on post-activation performance enhancement

**DOI:** 10.1371/journal.pone.0352993

**Published:** 2026-07-06

**Authors:** Alper Kartal, Muhammet Taha Ilhan, Esin Ergin, Zarife Pancar, Burak Karaca, Yasin Yildiz, Doğukan Batur Alp Gulsen, Serdar Bayrakdaroğlu

**Affiliations:** 1 Department of Coaching Education, Faculty of Sport Sciences, Aydın Adnan Menderes University, Aydın, Türkiye; 2 Department of Physical Education and Sports, Faculty of Sport Sciences, Gaziantep University, Gaziantep, Türkiye; 3 Department of Recreation, Faculty of Sport Sciences, Aydın Adnan Menderes University, Aydın, Türkiye; 4 Department of Coaching Education, Faculty of Sport Sciences, Gumushane University, Gumushane, Türkiye; Università degli Studi di Milano: Universita degli Studi di Milano, ITALY

## Abstract

The post-activation performance enhancement (PAPE) effect has been widely studied; however, studies conducted under real-world conditions remain limited. Furthermore, investigations into how different conditioning activities (CAs) influence PAPE responses are also scarce. Therefore, this study investigates the acute effects of high-intensity barbell hip thrust (BHT) and back squat (BS) exercises, performed after a soccer-specific warm-up (Wup), on physical performance in soccer players. Seventeen male amateur soccer players participated in the study (mean age: 22.29 ± 3.60 years; height: 1.78 ± 0.06 m; body weight: 73.18 ± 8.44 kg). Participants attended 5 experimental sessions. In the first session, after anthropometric assessments, participants were familiarized with the exercises and tests. In the second session, one-repetition maximum (1RM) was tested for BS and HT exercises. In the following three sessions, two PAPE protocols and one traditional (TRAD) protocol were performed in a randomized order. In the TRAD protocol, players performed physical performance tests including countermovement jump (CMJ), 10-m and 30-m sprint, and zigzag agility 16 minutes after a soccer-specific Wup. In the PAPE protocols, players performed CAs (e.g., hip thrust or back squat) at 85% of 1RM for 2 sets of 3 repetitions following a soccer-specific Wup, and completed the tests 8 minutes later. Results from the linear mixed-effects models revealed no significant effect of protocol on any performance variable, including CMJ (F(2,32) = 1.554, p = 0.227), 10 m sprint (F(2,32) = 0.242, p = 0.786), 30 m sprint (F(2,32) = 0.453, p = 0.640), and change of direction (COD) performance (F(2,32) = 0.946, p = 0.399). Marginal R² values indicated that the protocol explained only a minimal proportion of variance in performance outcomes (0.005–0.030), whereas conditional R² and ICC values suggested that between-participant differences accounted for a substantial proportion of variance, particularly for CMJ and 30 m sprint performance. Bonferroni-adjusted pairwise comparisons further showed no significant differences between PAPE_BHT_, PAPE_BS_, and TRAD protocols for CMJ, 10 m sprint, 30 m sprint, or COD performance (all p > 0.05). In conclusion, neither PAPE protocol performed after a soccer-specific warm-up significantly enhanced or impaired subsequent CMJ, sprint, or COD performance compared with the traditional warm-up. These findings suggest that adding PAPE_BHT_ or PAPE_BS_ protocols after a soccer-specific warm-up may maintain subsequent performance without inducing measurable decrements; however, their ergogenic effectiveness may depend on individual factors such as players’ strength levels, recovery capacity, training status, and physical characteristics.

## Introduction

Warm-up (Wup) routines are generally considered to be beneficial for performance [1, 2] and may also contribute to reducing injury risk, as demonstrated in applied sports settings [3–5]. In soccer, implementing an effective Wup protocol before training or matches is considered highly important for enhancing game performance [6]. Although the benefits of Wup are widely recognized, the optimal Wup strategy for soccer players prior to a match has yet to be fully established [7]. However, Wup strategies in soccer may include various types of exercises (or their combinations), such as static stretching [8, 9], dynamic stretching [8, 9], dynamic bodyweight exercises [10], or sport-specific exercises [11]. In addition to these exercises, a Wup strategy that includes post-activation performance enhancement (PAPE) has recently been introduced as an alternative to traditional Wup [12].

PAPE refers to acute increases in voluntary muscle performance resulting from pre-conditioning for high-intensity activities [13, 14]. While some studies have reported PAPE effects on physical performance—including vertical jump, sprint, and change of direction (COD)—following conditioning activity (CA) [15–18], others have shown no effect [19–22]. These conflicting findings may be attributed to differences in the PAPE protocols used (e.g., rest interval between CA and subsequent performance measurement, exercise intensity) [23–25], as well as to the characteristics of the participants included in the studies (e.g., strength level, resistance training experience) [26, 27]. For example, the magnitude of PAPE has been shown to increase with higher levels of maximal strength, with stronger individuals generally demonstrating greater performance improvements than weaker individuals [28, 29, 25]. An appropriate recovery interval following the conditioning activity is also critical, with rest periods of approximately 4–9 minutes reported to optimize performance [30, 31]. Exercise intensity plays a key role, as both moderate- and high-intensity loads can induce PAPE; however, stronger individuals may benefit more from higher intensities (≥ 85% 1RM), whereas weaker individuals tend to respond better to moderate loads (60–84% 1RM) [32, 33]. Proximity to muscular failure may further influence responses, as excessive fatigue can attenuate performance, while controlled effort levels may enhance outcomes [34, 35]. Finally, exercise selection within the conditioning activity may also influence PAPE responses, as different movements can produce varying performance outcomes [32].

In many studies examining the effects of PAPE, back squat (BS) exercise has been preferred as the CA [36, 37]. However, research on the effects of different CA is limited [23]. For example, the barbell hip thrust (BHT) exercise has recently been widely used to target the hip extensor musculature, including the gluteus maximus and hamstring muscles [38, 39]. Since BHT requires a consistent hip extension moment across the entire range of motion, it can effectively increase horizontal force production, improve sprint running speed, and induce gluteus maximus hypertrophy [39–42]. Despite these effects of BHT, only one study has investigated the PAPE effects of BHT as the CA in soccer players [17]. The findings of this study showed that BHT acutely improved soccer players’ sprint performance [17]. However, the absence of other studies may limit the use of this exercise as the CA. It is also known that the peak activity of the upper and lower gluteus maximus, as well as the biceps femoris, is higher during BHT compared to BS [43]. These different neuromuscular properties may produce varying PAPE effects. Yet, to the authors’ knowledge, no study in the literature has compared the PAPE effects of these two exercises when used as CAs.

Moreover, since many studies investigating the effects of PAPE have been conducted in laboratory settings, the impact of PAPE strategies under real-world conditions remains uncertain [13, 23]. Therefore, investigating the effects of PAPE strategies involving HT and BS in real-world settings may provide valuable insights. Soccer competition rules require the pre-match Wup to be completed 15–20 minutes before the start of the game. Considering that this period following the Wup is often spent in passive rest, which may negatively affect players’ physical performance [44], implementing PAPE strategies during this interval may help maintain or even enhance the positive effects of the Wup. Therefore, the aim of this study is to investigate the acute effects of high-intensity BHT and BS exercises, performed after a soccer-specific Wup, on male soccer players’ jumping, sprinting, and COD performance.

## Methods

### Experimental approach to the problem

**Fig 1:**
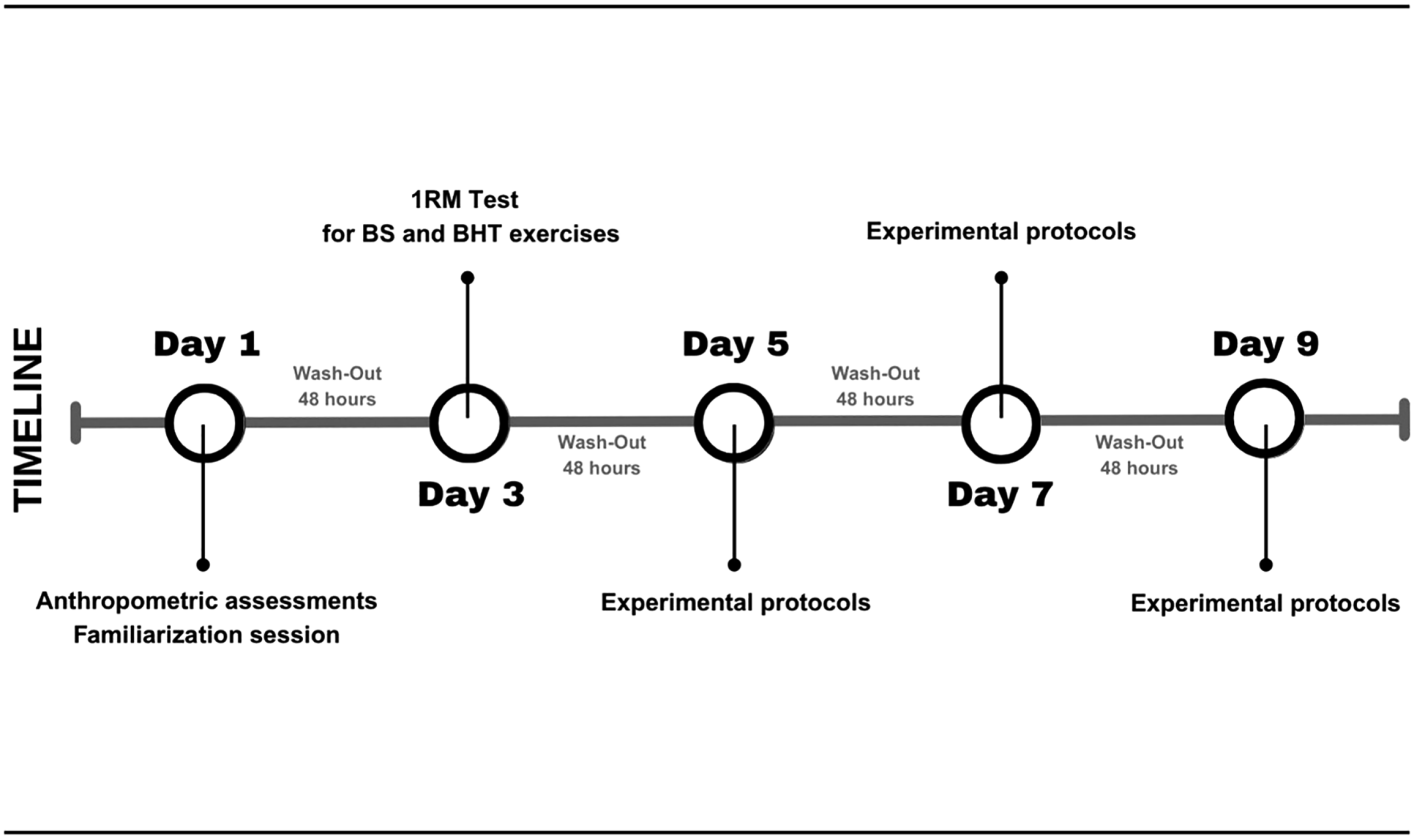
Study design. 1RM: One-repetition maximum; BS: Back squat; BHT: Barbell hip thrust.

**Fig 2:**
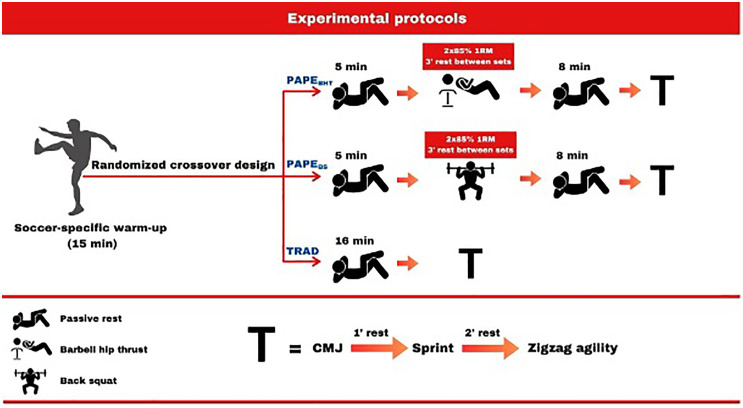
Experimental protocols. PAPE_BHT_: Post-activation performance enhancement protocol involving the barbell hip thrust exercise; PAPE_BS_: Post-activation performance enhancement protocol involving the back squat exercise; TRAD: approach involving the execution of tests 16 minutes after a standard soccer-specific warm-up; 1RM: One-repetition maximum; CMJ: Countermovement Jump.

This study was conducted using a randomized crossover design (Fig 1). The study involved seventeen male soccer players. The study comprised five sessions, each separated by a minimum interval of 48 hours to ensure adequate recovery and data reliability. In the first session, following the measurement of players’ height and body mass, a familiarization phase was carried out to introduce the exercises and tests to be used in the study. In the second session, the one-repetition maximum (1RM) of the players was assessed for the BS and HT exercises. In the following three sessions, three experimental protocols were performed in a randomized order (Fig 2): (1) The PAPE protocol (PAPE_BHT_) involved performing 2 sets of 3 repetitions of the BHT exercise at 85% of 1RM, 5 minutes after the standard soccer-specific Wup, followed by testing after an 8-minute passive rest; (2) The PAPE protocol (PAPE_BS_) involved performing 2 sets of 3 repetitions of the BS exercise at 85% of 1RM, 5 minutes after the standard soccer-specific Wup, followed by testing after an 8-minute passive rest; (3) The traditional (TRAD) approach involving the execution of tests 16 minutes after a standard soccer-specific Wup. The PAPE protocol was designed based on recommendations from previous studies to optimize the PAPE response, particularly with respect to recovery interval, exercise intensity, and conditioning activity selection [23–25,32]. In addition, the recovery intervals were structured to reflect real-world match scenarios, specifically the typical time window between the completion of a soccer-specific warm-up and the onset of competition.

The players performed countermovement jump (CMJ), 10-m and 30-m sprint, and zigzag agility tests in all protocols. These tests were conducted on artificial turf except for the CMJ, which was performed indoors. All assessments were performed at the same time of day (12:00–16:00) and under similar temperature conditions (25–29°C). Players were instructed to avoid caffeine consumption and any other resistance training for 48 hours prior to the assessments.

### Participants

The sample size was determined through an a priori power analysis using GPower software (GPower, version 3.1.9.7, University of Düsseldorf, Düsseldorf, Germany). The analysis was performed for a repeated-measures ANOVA (within factors) with one group and three measurements (conditions), in which all participants completed each experimental protocol, using the following parameters: effect size f = 0.40, α err prob = 0.05, power (1 − β err prob) = 0.80, correlation among repeated measures = 0.50, and nonsphericity correction ε = 1. The selected effect size was based on previous research reporting moderate effects of PAPE on performance outcomes [13]. Based on this analysis, the recommended minimum sample size was determined to be 12 participants. However, 17 participants were included in the study to increase statistical power and account for potential dropouts (Table 1).

**Table 1:** Descriptive statistics of the subjects.

Variables	Mean ± SD	95% Confidence Interval for Mean	
		Lower Bound	Upper Bound
Age (year)	22.29 ± 3.60	20.44	24.15
Body height (m)	1.78 ± 0.06	1.75	1.81
Body mass (kg)	73.18 ± 8.44	68.84	77.52
BMI (kg/m^2^)	23.06 ± 1.84	22.11	24.01
1RM BHT (kg)	155.29 ± 27.64	141.08	169.51
1RM BS (kg)	114.71 ± 14.63	107.18	122.23
Relative BHT (1RM/body mass)	2.13 ± 0.37	1.94	2.32
Relative BS (1RM/body mass)	1.58 ± 0.26	1.45	1.72
Soccer training experience (year)	12.12 ± 4.39	9.86	14.37
Resistance training experience (year)	5.06 ± 1.95	4.06	6.06

SD: Standard deviation; BMI: Body mass index; 1RM: One-repetition maximum; BHT: Barbell hip thrust; BS: Back squat.

Participants were recruited through announcements made at the university, constituting a convenience sample. All participants were actively competing in organized local amateur soccer leagues at a similar competitive level and can be classified as sub-elite players, with regular training and official match participation throughout the competitive season. Moreover, the inclusion criteria were as follows: (a) absence of musculoskeletal injuries or other health conditions that would prevent participation in the study; (b) engagement in resistance training at least three days per week for a minimum of one year; and (c) being a male soccer player aged between 18 and 35 years. Additionally, all players participated in regular soccer training sessions lasting approximately 90 minutes, four days per week.

The benefits and risks of the study were explained to the players, and written informed consent was obtained from them. The study commenced after obtaining ethical approval, as per protocol number 2024/21 and decision number 10 from the Non-Interventional Clinical Research Ethics Committee of Aydın Adnan Menderes University Faculty of Medicine. In accordance with the Declaration of Helsinki, all participants provided written informed consent after being informed about the study protocol. The recruitment period for participants was conducted between 08/04/2024 and 21/04/2024.

## Procedures

### Post-activation performance enhancement protocol

A soccer-specific standardized 15-minute Wup was performed prior to all experimental protocols. Following the Wup, a 5-minute passive rest period was provided. After the rest period, the PAPE protocols were carried out. Resistance exercises in the PAPE protocols were performed for 2 sets of 3 repetitions at 85% of 1RM, with a 3-minute rest between sets. After the second set was completed, an 8-minute passive rest period was provided, followed by the CMJ, sprint, and COD tests performed in sequence (Fig 2).

The back squat (BS) and barbell hip thrust (BHT) exercises were performed in accordance with previously described techniques. For the BS, participants positioned the barbell across the upper back and descended until the thigh reached at least parallel to the ground (≈90° knee flexion), while maintaining an upright trunk and neutral spine. Knees were aligned with the toes throughout the movement to avoid valgus collapse [45]. For the BHT, participants positioned their upper back against a padded bench (just below the scapulae) with a barbell placed over the pelvis. Feet were shoulder-width apart, with the tibia perpendicular to the ground at the top position. The movement was performed through hip extension by contracting the gluteal muscles while maintaining a neutral spine. At the top position, the trunk was parallel to the ground before lowering the barbell in a controlled manner [39]. In both exercises, movements were performed with a controlled eccentric phase, and participants were instructed to perform the concentric phase as fast as possible while maintaining proper technique.

### Soccer-specific warm-up

The soccer-specific Wup protocol was conducted in accordance with the recommendations of previous literature [46]. Details of the protocol are provided in Table 2.

**Table 2:** Characteristics of the soccer-specific warm-up protocol.

Content	Time (15 min)
Aerobic work and joint mobility (min)	0-2
Individual and collective technical exercises (min)	2-6
Free circulation with the ball (min)	2-3
Pass (min)	3-4
Ball driving (min)	4-5
Possession 4 vs.1 (min)	5-6
Static stretching (min)	6-7
Dynamic stretching (min)	7-8
Small sided game 5 vs. 5 (min)	8-9
Break (min)	9-10
Small sided game 5 vs. 5 (min)	10-11
Long passes (min)	11-13
Sprint starts (min)	13-15

### Anthropometric measures

Height was measured using a stadiometer (SECA, Hamburg, Germany) with an accuracy of 0.1 cm. Body mass was measured using a body scale (Fakir, Germany). Both measurements were conducted in the morning prior to breakfast. After the measurements, body mass index (BMI) was calculated using the height and body mass values.

### One-repetition maximum test

The 1RM measurements for the BS and HT exercises were performed according to the protocol previously established by Brown and Weir [47]. After a standardized Wup, players performed 8 repetitions of the exercise at approximately 50% of their estimated 1RM, followed by 3 repetitions at approximately 70% of their estimated 1RM. After that, the lifts were performed as single repetitions and continued until the player was unable to proceed. This procedure continued until the 1RM was established. A 3-minute rest period was provided between trials. The 1RM was determined within a maximum of 5 trials. After determining the 1RM for the BS exercise, a 10-minute rest period was provided, followed by the 1RM test for the HT exercise. The exercises were performed with an Olympic barbell.

### Vertical jump test

Vertical jump performance was evaluated through the CMJ test, which was performed with a small pressure-sensitive contact mat (Smart Speed; Fusion Sport, Brisbane, Australia) at an indoor sports facility. The CMJ test was performed with the subject standing upright and hands placed on the hips. During this test, the athletes were instructed to avoid swinging their arms. The player’s downward movement was immediately followed by an upward vertical movement. Participants were verbally instructed to jump as high as possible while maintaining an upright posture and keeping their hands on their hips throughout the movement. The test was performed twice with a 30-second interval, and the highest jump was used for analysis [48].

### 10- and 30-m sprint test

One minute after the CMJ test, players performed two maximal 30-meter sprints. Sprint performance was measured using photocell timing gates (Newtest, Oy, Finland) positioned at 10 m and 30 m from the starting line. A 2-minute recovery period was provided between sprints. Participants were verbally instructed to perform each sprint with maximal effort from a standing start while maintaining proper running technique. The fastest time was used for analysis [49].

### Change of direction test

**Fig 3:**
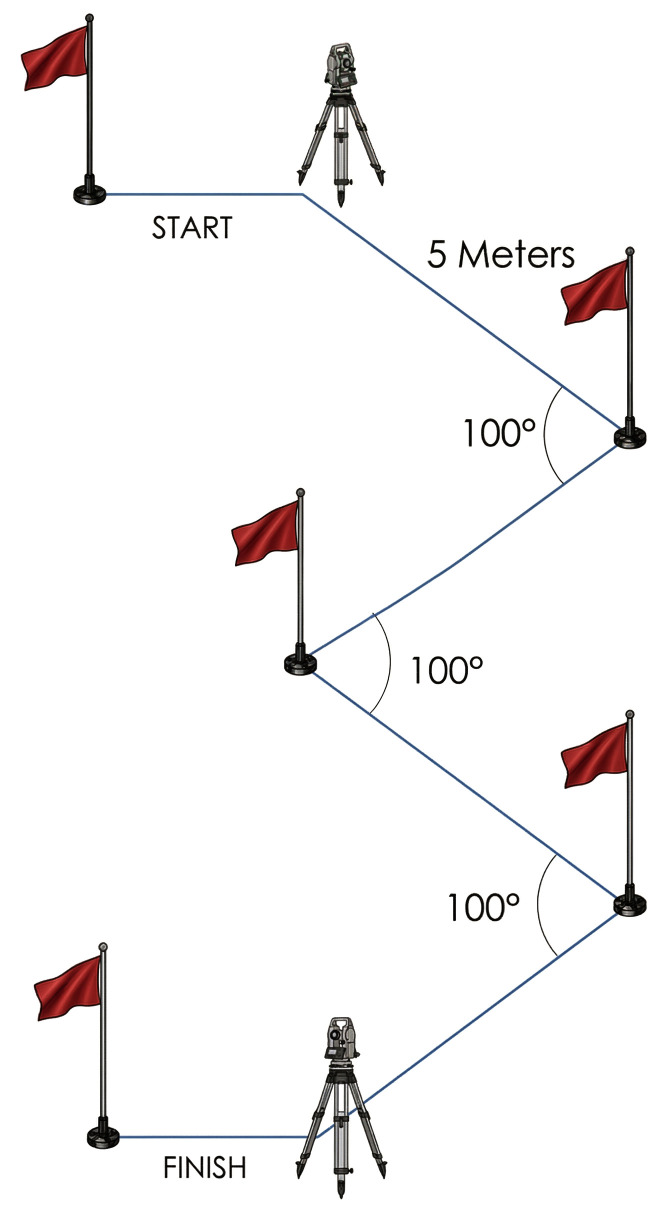
Schematic representation of the zig-zag agility test course used in the study.

COD ability was evaluated using the zigzag agility test without a ball, which was performed 2 minutes after the sprint test. The test consisted of four 5-meter sections arranged at an angle of 100°. This test is based on rapid acceleration, deceleration, re-acceleration, and balance control, all of which are required for short-distance running performance [50]. Participants were instructed to complete the course as quickly as possible while maintaining control and proper footwork during changes of direction. Two trials were performed with a 1-minute rest period between them. The best result was selected for analysis (Fig 3).

### Statistical analysis

SPSS package program (SPSS for Windows, version 22.0, SPSS Inc., Chicago, Illinois, USA) was used to statistically analyze the data obtained at the end of the study. Data were presented as mean and standard deviation. The Shapiro-Wilk test was used to assess normality, and Levene’s test was used to evaluate homogeneity of variance. Skewness and kurtosis values were also checked, and values within ±2 were considered to indicate acceptable normal distribution [51]. Statistical significance was set at p < 0.05.

To account for inter-individual variability and the repeated-measures nature of the data, linear mixed-effects models were conducted. Protocol was included as a fixed effect, while participant was included as a random intercept. Pairwise comparisons between protocol conditions were performed using Bonferroni adjustment. Model fit was evaluated using marginal and conditional R² values. In addition, the intraclass correlation coefficient (ICC) was calculated to quantify the proportion of variance attributable to between-participant differences.

## Results

Linear mixed-effects model analyses revealed no significant effect of protocol on any of the performance variables, including CMJ (F(2,32) = 1.554, p = 0.227), 10 m sprint (F(2,32) = 0.242, p = 0.786), 30 m sprint (F(2,32) = 0.453, p = 0.640), and COD (F(2,32) = 0.946, p = 0.399). The marginal R² values indicated that protocol explained a minimal proportion of variance across all variables (CMJ = 0.014, 10 m = 0.009, 30 m = 0.005, COD = 0.030), whereas conditional R² values were higher for CMJ (0.771) and 30 m sprint (0.705), and lower for 10 m sprint (0.023) and COD (0.201). The intraclass correlation coefficients (ICC) were notably high for CMJ (0.768) and 30 m sprint (0.703), and lower for COD (0.176) and 10 m sprint (0.014) (Table 3).

**Table 3:** Linear mixed-effects model results for the effects of protocol on CMJ, sprint, and COD performance variables.

Variable	F (df)	p	Marginal R²	Conditional R²	ICC
CMJ	1.554 (2,32)	0.227	0.014	0.771	0.768
10 m	0.242 (2,32)	0.786	0.009	0.023	0.014
30 m	0.453 (2,32)	0.640	0.005	0.705	0.703
COD	0.946 (2,32)	0.399	0.030	0.201	0.176

A linear mixed-effects model with participant included as a random intercept was used for all analyses. CMJ: Countermovement jump; COD: Change of direction; PAPE_BHT_: hip thrust protocol; PAPE_BS_: back squat protocol; TRAD: traditional warm-up. Marginal R² represents the variance explained by fixed effects, whereas conditional R² includes both fixed and random effects. The intraclass correlation coefficient (ICC) reflects the proportion of total variance attributable to between-participant differences.

**Fig 4:**
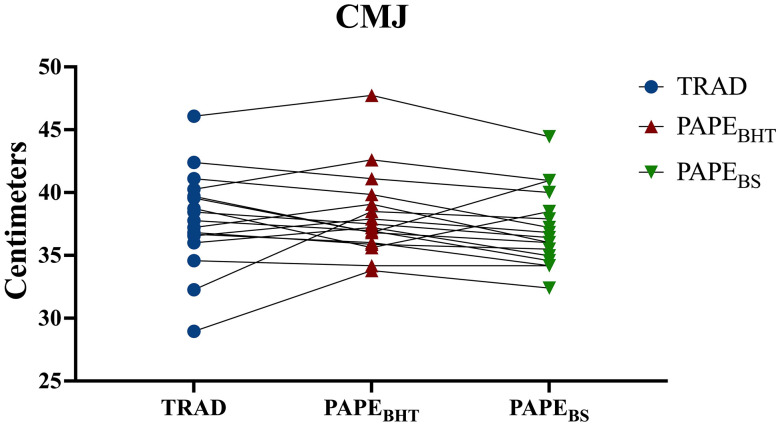
Effects of the protocols on CMJ. CMJ: Countermovement jump; TRAD: Traditional; PAPE_BHT_: Post-activation performance enhancement protocol involving the barbell hip thrust exercise; PAPE_BS_: Post-activation performance enhancement protocol involving the back squat exercise.

**Fig 5:**
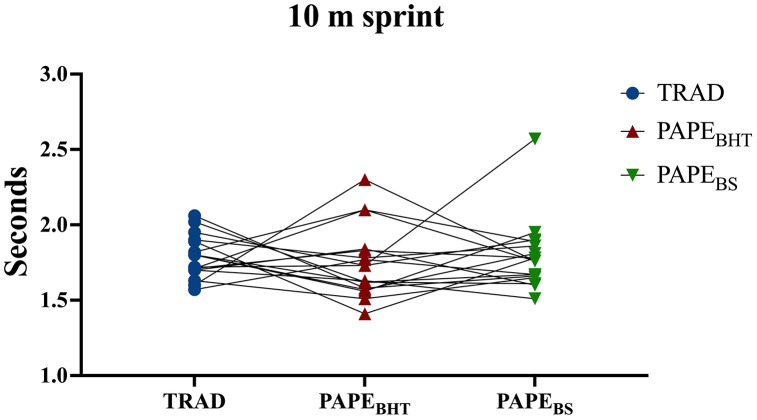
Effects of the protocols on 10 m sprint. TRAD: Traditional; PAPE_BHT_: Post-activation performance enhancement protocol involving the barbell hip thrust exercise; PAPE_BS_: Post-activation performance enhancement protocol involving the back squat exercise.

**Fig 6:**
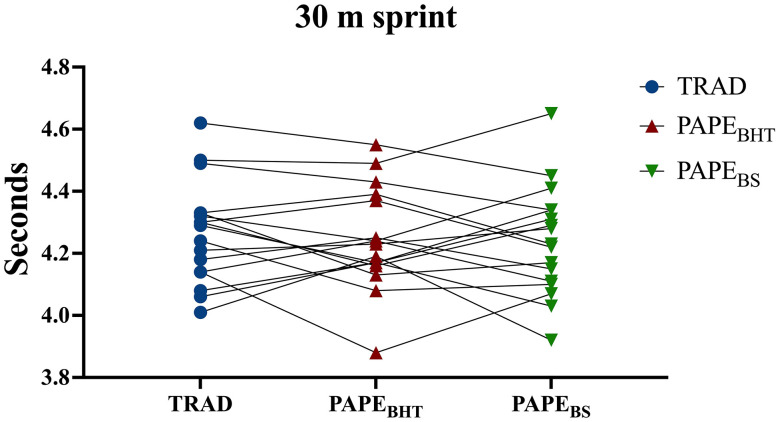
Effects of the protocols on 30 m sprint. TRAD: Traditional; PAPE_BHT_: Post-activation performance enhancement protocol involving the barbell hip thrust exercise; PAPE_BS_: Post-activation performance enhancement protocol involving the back squat exercise.

**Fig 7:**
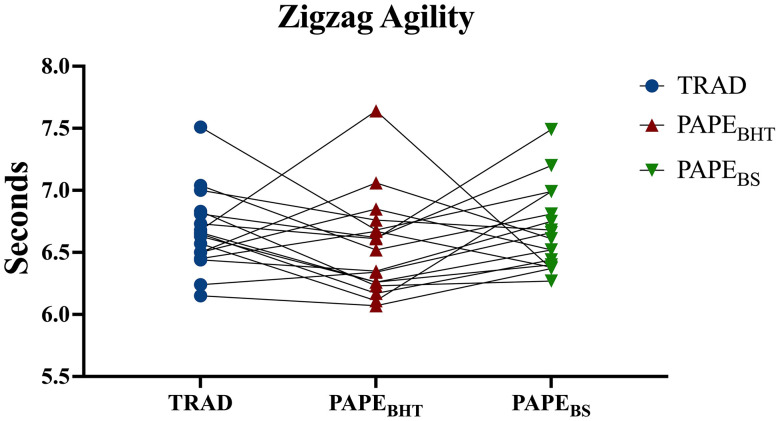
Effects of the protocols on change of direction (COD) performance. TRAD: traditional warm-up; PAPE_BHT_: post-activation performance enhancement protocol involving the barbell hip thrust exercise; PAPE_BS_: post-activation performance enhancement protocol involving the back squat exercise.

Pairwise comparisons between protocol conditions indicated no statistically significant differences for any of the performance variables (all p > 0.05). For CMJ, no significant differences were observed between PAPE_BHT_ and TRAD (β = 0.262, p = 0.650), PAPEBS and TRAD (β = −0.711, p = 0.222), or PAPE_BHT_ and PAPE_BS_ (β = 0.972, p = 0.098). Similarly, for 10 m sprint, PAPE_BHT_ vs TRAD (β = −0.041, p = 0.565), PAPE_BS_ vs TRAD (β = 0.003, p = 0.967), and PAPE_BHT_ vs PAPE_BS_ (β = −0.044, p = 0.538) comparisons were not significant. For 30 m sprint, PAPE_BHT_ vs TRAD (β = −0.024, p = 0.458), PAPE_BS_ vs TRAD (β = −0.028, p = 0.384), and PAPE_BHT_ vs PAPE_BS_ (β = 0.004, p = 0.896) showed no significant differences. Likewise, no significant differences were found in COD performance between PAPE_BHT_ and TRAD (β = −0.129, p = 0.249), PAPE_BS_ and TRAD (β = 0.004, p = 0.975), or PAPE_BHT_ and PAPE_BS_ (β = −0.132, p = 0.236) (Table 4) (Figs 4–7).

**Table 4:** Pairwise comparisons between protocols for CMJ, sprint, and COD performance variables based on linear mixed-effects models.

Variable	Effect / Comparison	Estimate (β)	SE	95% CI	t	df	p
**CMJ**	Intercept	37.680	0.770	36.130–39.231	48.924	16	< 0.001
	PAPE_BHT_ vs TRAD	0.262	0.571	−0.887–1.411	0.459	32	0.650
	PAPE_BS_ vs TRAD	−0.711	0.571	−1.859–0.438	−1.245	32	0.222
	PAPE_BHT_ vs PAPE_BS_	0.972	0.571	−0.190–2.135	1.704	32	0.098
**10 m Sprint**	Intercept	1.774	0.030	1.715–1.834	60.096	16	< 0.001
	PAPE_BHT_ vs TRAD	−0.041	0.071	−0.184–0.101	−0.581	32	0.565
	PAPE_BS_ vs TRAD	0.003	0.071	−0.140–0.146	0.042	32	0.967
	PAPE_BHT_ vs PAPE_BS_	−0.044	0.071	−0.188–0.100	−0.623	32	0.538
**30 m Sprint**	Intercept	4.250	0.036	4.177–4.323	116.659	16	< 0.001
	PAPE_BHT_ vs TRAD	−0.024	0.031	−0.087–0.040	−0.751	32	0.458
	PAPE_BS_ vs TRAD	−0.028	0.031	−0.091–0.035	−0.882	32	0.384
	PAPE_BHT_ vs PAPE_BS_	0.004	0.031	−0.060–0.068	0.131	32	0.896
**COD**	Intercept	6.628	0.057	6.513–6.744	115.550	16	< 0.001
	PAPE_BHT_ vs TRAD	−0.129	0.110	−0.349–0.092	−1.175	32	0.249
	PAPE_BS_ vs TRAD	0.004	0.110	−0.217–0.224	0.032	32	0.975
	PAPE_BHT_ vs PAPE_BS_	−0.132	0.110	−0.356–0.091	−1.207	32	0.236

Pairwise comparisons between protocols were conducted within the linear mixed-effects framework and adjusted using Bonferroni correction. Values represent estimated mean differences between conditions with corresponding standard errors and 95% confidence intervals. Confidence intervals including zero indicate a lack of statistically significant difference between protocols.

## Discussion

This study aims to investigate the acute effects of the PAPE_BHT_ and PAPE_BS_ protocols, conducted after a soccer-specific Wup, on jumping, sprinting, and COD performance in male soccer players. The findings indicate that both PAPE protocols performed following a soccer-specific Wup did not have a significant effect on subsequent physical performance.

We did not find any improvement in CMJ height and sprint performance following high-intensity BHT and BS conditioning activities. A recent systematic review and meta-analysis reported that PAPE protocols are not effective in enhancing vertical jump performance and that the existing studies have produced conflicting findings [30]. Indeed, when examining studies on vertical jump performance, some have found a PAPE effect following CA [13, 52, 53], while others have not observed such an effect [19–21]. Studies examining the effects of PAPE protocols on sprint performance have also reported conflicting findings [15–17, 20, 21, 53]. There are very few studies investigating PAPE responses in terms of COD performance. While some studies have reported a PAPE effect [13, 18], Marshall et al. [22] did not observe such an effect. These inconsistent findings may be influenced by factors such as participants’ strength levels, resistance training experience, the type of CA, and the recovery interval between the CA and subsequent performance [37, 54]. Maloney et al. [18] reported that performance enhancement emerged after 4–6 minutes of recovery, whereas Abade et al. [13] observed improvements as early as 1 minute post-CA in athletes with high relative strength levels. In contrast, Marshall et al. [22] found no improvement in COD performance following an isometric and biomechanically less specific CA, with fatigue effects dominating, particularly in the early recovery phase, suggesting that the applied recovery intervals may not have been sufficient to elicit potentiation. These findings suggest that PAPE responses depend not only on strength level but also on the biomechanical specificity of the CA and the optimization of recovery duration. Stronger individuals may exhibit earlier and greater potentiation responses, whereas weaker individuals may require longer recovery periods [54, 37]. However, variations in protocols across studies (i.e., type of CA, participant characteristics, and recovery intervals) limit the generalizability of findings and make it difficult to draw definitive conclusions regarding the effectiveness of PAPE protocols on COD performance.

Faster sprinters and stronger athletes typically possess a higher proportion of fast-twitch (Type II) muscle fibers. These fibers become more active during high-speed and forceful movements, enabling short-term, high-power output. Wilson et al. [26] reported that these fibers play a crucial role in the PAPE response because their motor neuron excitability is increased by the central nervous system during PAPE. Yetter and Moir [55] explain that, as a result of this stimulation, fast-twitch fibers are more activated, which may enhance the PAPE effect. In stronger and faster athletes, the PAPE effect may be more pronounced depending on the density of these fibers. For example, a stronger sprinter may demonstrate improved speed or jump performance due to the increased excitability of the central nervous system following the CA. This suggests that the PAPE effect is influenced by individual differences. Indeed, Villalon-Gasch et al. [56] demonstrated that the effects of PAPE depend on various individual factors such as athletes’ physiological characteristics, experience, age, muscle fiber distribution, and training level. In the study conducted by Jarosz et al. [57], following a maximal isometric squat CA, a PAPE effect on CMJ height was observed in 8 out of 15 participants, no effect was seen in 5 participants, and a performance decline was detected in 2 participants. Similarly, Till and Cooke [20] reported that PAPE causes large variations in individual responses, ranging from −7.11% to +8.2%. Therefore, PAPE protocols should be designed with individualization in mind. However, the level of competition may play an important role in the presence of the PAPE effect [58]. Bishop [59] stated that potential neuromuscular and energy-related fatigue effects following Wup involving PAPE may be low in well-trained athletes. Although the players in our study had high training experience (12.12 ± 4.39 years), their level of competition was low (amateur). Therefore, the CA performed after the soccer-specific Wup may have increased fatigue in the players.

In our study, the participants’ low strength level for the BS exercise (relative 1RM BS = 1.58 ± 0.26) may have contributed to the absence of PAPE responses. In the study conducted by Sañudo et al. [27], participants were divided into two groups—strong (relative 1RM back squat > 2.0) and weak (relative 1RM back squat < 2.0)—for analysis. The study’s findings concluded that only the strong participants showed improvements in sprint performance. However, in the present study, the lack of PAPE effect during sprint performance may be attributed to several physiological factors. First, the central nervous system may not fully recover after a heavy-load CA, which could limit motor neuron excitability and prevent sufficient activation of fast-twitch muscle fibers. Secondly, intense muscle fatigue and limitations in energy systems—particularly insufficient replenishment of the ATP-PCr system—may restrict optimal power output during sprinting. Finally, the inability to tailor recovery time to individual needs may suppress the PAPE effect and negatively impact sprint performance. Recovery time is a critical factor influencing the PAPE effect, as it determines the balance between fatigue and potentiation [29]. Previous studies have generally suggested that the optimal recovery interval ranges between 5 and 10 minutes [25, 32, 54], although shorter or individualized intervals may also be effective depending on the protocol and participant characteristics [60–62, 28]. These findings indicate that recovery duration should be individualized when examining PAPE responses following CA.

This study has several limitations that should be taken into account when interpreting the findings. Firstly, the sample consisted of a relatively small and homogeneous group of male amateur soccer players, which may limit the generalizability of the results to other populations, such as professional players or female athletes. Secondly, the study did not include any measurement of neuromuscular fatigue or muscle activation (e.g., EMG analysis), which could have provided additional insight into the mechanisms underlying PAPE effects. Thirdly, the rest interval between the CA and performance tests was standardized at eight minutes for all participants. However, individual differences in optimal rest time might have influenced the PAPE response. Fourthly, the soccer-specific Wup may represent an additional limitation, as it may have influenced the PAPE response. A comprehensive and task-specific Wup may have already elicited physiological responses similar to those underlying PAPE (e.g., increased muscle temperature and blood flow) [23], potentially leading participants to reach a performance plateau and limiting any additional effect of the subsequent CA [22]. In addition, the intensity and duration of the Wup may have induced fatigue, shifting the balance between fatigue and potentiation toward fatigue and thereby attenuating the PAPE response [22]. This may partly explain the lack of performance enhancement observed in the present study. Finally, the study focused on acute performance effects rather than longer-term adaptations, so it remains unclear how repeated exposure to the experimental protocols might impact performance outcomes over time. To the best of our knowledge, this is the first study to investigate the effects of heavy-load BHT and BS conditioning activities performed after soccer-specific Wup on the physical performance of soccer players, despite these limitations. Future research could consider implementing similar protocols with different age groups, female athletes, and players from various levels of competition to enhance the generalizability of the findings. Additionally, exploring individualized optimal rest intervals and different strength protocols may provide deeper insights into the underlying mechanisms of PAPE. Incorporating measures of neuromuscular fatigue and biochemical parameters (e.g., lactate, creatine kinase) would also help to elucidate the physiological responses to PAPE protocols in soccer players. However, future studies may help to better understand the effects of PAPE on athletes by examining different exercise protocols and recovery times.

## Conclusion

In conclusion, this study indicates that both PAPE protocols performed following a soccer-specific Wup did not have a significant effect on subsequent vertical jump, sprint, and COD ability performances. These findings emphasize the importance of tailoring PAPE protocols to individual athletes, especially in sports like soccer. Coaches should consider players’ strength levels and recovery profiles when planning pre-match Wup, as these factors may influence the effectiveness of PAPE interventions. Implementing individualized Wup strategies may help optimize performance responses.

## Supporting information

S1 Dataset Raw dataset used for all statistical analyses. (XLSX)

S2 Dataset Participant characteristics. (XLSX)

## Abbreviations

1RM: One-repetition maximum
BHT: Barbell hip thrust
BMI: Body mass index
BS: Back squat
CA: Conditioning activity
CMJ: Countermovement jump
PAPE: Post-activation performance enhancement
PAPE_BHT_: Post-activation performance enhancement protocol involving the barbell hip thrust exercise
PAPE_BS_: Post-activation performance enhancement protocol involving the back squat exercise
TRAD: Approach involving the execution of tests 16 minutes after a standard soccer-specific warm-up
Wup: Warm-up
